# Child sexual abuse and disclosure in South Western Nigeria: a community based study

**DOI:** 10.4314/ahs.v18i2.2

**Published:** 2018-06

**Authors:** Nkiruka David, Oliver Ezechi, Agatha Wapmuk, Titilola Gbajabiamila, Aigbe Ohihoin, Ebiere Herbertson, Kofoworola Odeyemi

**Affiliations:** 1 Nigerian Institute of Medical Reserach, Clinical Sciences Department; 2 University of Lagos

**Keywords:** Adolescent, child, sexual abuse

## Abstract

**Introduction:**

The true burden of child sexual abuse in Nigeria is not known as most reports are institutional based. This study was designed to determine population level data on the burden and pattern of child sexual abuse among adolescents in South Western Nigeria.

**Methods:**

A community based study among adolescents in SouthWestern Nigeria. Semi- structured interviewer administered questionnaire was used to collect data from respondents selected through a multistage sampling technique. Analysis was with SPSS version 20.

**Results:**

398 adolescents (314 females and 84 males) aged 10–19 years with a mean age of 15.6 ± 2.0 years participated in the study. Most were single (90.7%), in school (84.2%), and lived with their parents/guardians (89.4%). The prevalence of CSA was 25.7%. Penetrative abuse occurred in 7.5%, and forced abuse in 46.2% of cases. Perpetrators were mostly boyfriends (31.2%) and neighbours (16.1%). Intra-familial abuse occurred in 7.5% of cases. Only 34.4% of cases ever disclosed the abuse.

**Conclusion:**

CSA is common in the community, with perpetrators majorly persons known to the adolescents. A large number of cases are not reported. Efforts should be made to educate children and their parents on various ways to reduce child sexual abuse and its consequences.

## Introduction

Sexual abuse of children is the ‘involvement of a child in sexual activity that he or she does not understand, cannot give informed consent to, or for which the child is not developmentally prepared, or that violates the laws or social taboos of society’.^1^ It is a violation of the rights of the child and is a social and public health concern that occurs in every country in the world across diverse social, economic and racial groups. The spectrum of activities comprising child sexual abuse (CSA) includes indecent exposure of genitals to a child, inappropriate touch, and forced sexual intercourse (rape). The exploitative use of a child in prostitution or other unlawful sexual practices is also a form of child sexual abuse.^2^,^3^

Globally the prevalence of child sexual abuse ranges from 5%–36%.^4^–^8^ Studies in sub-Saharan Africa have also shown similar prevalence rates.^9^–^11^ Although the true burden of CSA in Nigeria is unknown, it is estimated to vary between 5% and 38% across different parts of the country.^12^–^15^ The true magnitude of the problem however remains difficult to determine. This is as a result of the culture of silence around the issue of child sexual abuse, especially in the African setting where sexual matters are not discussed in public. Non-disclosure of abuse by the victims, especially the very young, arising from fear of further harm from the perpetrator or of being blamed; feelings of shame, and cultural inhibitions also help to mask the true burden of CSA. Ineffectual prosecution of offenders in reported cases also contributes to non-disclosure of abuse.^1^,^3^

Child sexual abuse appears to be more common in females than males, usually occurs in settings the child is familiar with, and most abusers are known to the children before the onset of the abuse.^1^,^16^ Circumstances associated with such abuse include poverty, ignorance, poor education, and unstable home environments.^11^,^16^ In the African setting, some of these factors are enhanced by the erosion of cultural values and the extended family safety nets, as a result of rapid urbanization and westernization.^17^

Adverse physical and psychological consequences of CSA include physical trauma, acquisition of sexually transmitted infections including HIV, teenage pregnancy and its associated adverse sequelae, development of anti-social behavior, and inability to have a satisfying sexual relationship in the future. These consequences have negative impact not only on the child but also on the family and the society at large.^3^

Adolescent age group is characterized by morphologic changes, increasing self-awareness and risk taking behavior, but until quite recently was often neglected in health planning. The health and social issues of adolescents needs to be highlighted especially as most often they are population commonly abused among the children but rarely reported.^12^–^14^ In addition, there is a dearth of information on the true burden of adolescent child sexual abuse in Nigeria as the few available studies have been in institutional settings. The present study was designed to obtain data from the community with the aim of determining the prevalence of, and factors associated with, child sexual abuse in adolescents in Mushin, a cosmopolitan community in Lagos State, SouthWest Nigeria.

## Methods

This was a cross-sectional community based study conducted in Mushin, a densely populated community in Lagos State, the former administrative capital and commercial nerve centre of Nigeria, with an estimated population of about 21 million persons.^18^ Data was collected between September and November 2014.

### Study population

Male and female in-school and out-of-school adolescents aged 10–19 years who were resident in the community constituted the study population. The study sample size of 362 was obtained using the formula for cross-sectional studies,^19^ and a prevalence of 38%^15^ and error margin of 5%. The sample size was increased to 398 as 10% was added to take care of poorly filled questionnaires.

### Sampling method

A multistage sampling technique was used to select participants for the study. Three wards identified as AL, BA and AT were randomly selected from the 10 wards in the community. There are 68, 32 and 42 streets in the wards respectively in the three selected wards. The study population recruited from AL, BA and AT ward was 190, 88 and 120 respectively in the proportion of 68: 32: 42 or 0.48: 0.22: 0.30 among wards. Streets were randomly picked till the selected no of participants per ward was achieved. In each of the selected streets the starting house was randomly selected from the total list of house numbers on that street. Thereafter consecutive residential houses were used. In each house, one adolescent who met the study eligibility criteria was consented for inclusion into the study. If there were more than one such adolescent, one was randomly selected for inclusion into the study after the informed consent process. This process continued until the required number of study participants per ward was recruited.

### Data management

Field assistants were recruited and trained to administer the questionnaire to the study participants. Data was collected from consenting eligible participants over a twomonth period, on Thursdays, Fridays and Saturdays. This was done to ensure that both the in-school and out-ofschool adolescents in Mushin Community were captured in the study. A semi-structured, interviewer-administered questionnaire was used to collect data on socio-demographic characteristics; and history, and circumstances of child sexual abuse as applicable.

Information from completed questionnaires was entered into a Statistical Package for Social Sciences (SPSS) spread sheet and analyzed using SPSS version 20. Frequencies of characteristics of study participants were generated and Chi square test was used to analyze the categorical variables. Statistical significance was set at p value less than 0.05.

### Ethical consideration

Ethical approval for the study was obtained from the Health Research Ethics Committee (HREC) of the Lagos University Teaching Hospital (LUTH), Idi-Araba, and Nigerian Institute of Medical Research, Lagos. The study and its processes were explained to the prospective participants and their parents/caregivers (if they were less than 18 years). Informed consent (from participants aged ≥ 18 years and parents/guardians of participants aged less than 18 years), and assent (from participants aged <18 years) were obtained as necessary.

## Results

A total of three hundred and ninety eight adolescents were interviewed during the study period. Female adolescents were the majority (Females: 314; 78.9%; Males: 84; 21.1%), giving a female: male ratio of 3.7: 1. The mean age of respondents was 15.6 ± 2.0 years (males: 15.5 ± 2.1 years; females: 15.6 ± 2.0 years). Majority of participants were single (361; 90.7%), Muslims (209;52.5%), in school (335; 84.2%), lived with their parents or guardians (356; 89.4%), and lived in one or two rooms with shared conveniences (228; 57.3%). Majority of the parents of the respondents were living together (277; 69.6%), had at least secondary school education (331; 83.2%) and 316; 79.4%) for fathers and mothers respectively), and were traders or artisans (165; 41.5%) and 253; 63.6%) for fathers and mothers respectively). Major reason (in 34 of 63; 53.9% cases) for dropping out of school for the out-of-school participants was financial constraints. The sociodemographic and parental characteristics of participants are illustrated in table 1.

**Table 1:** Socio-demographic and parental characteristics of participants

Variable (N =398)	Frequency (%)
**Sex:**	
Male	84 (21.1)
Female	314 (78.9)
**Marital Status:**	
Single	361 (90.7)
Married	20 (5.0)
Cohabiting with Partner	9 (2.3)
Separated/Divorced	8 (2.0)
**In School:**	
Yes	335 (84.2)
No	63 (15.8)
**Accommodation**	
One/two rooms (shared conveniences)	228 (57.3)
Self-contained Apartment/House	170 ( 42.7)
**Parent Marital Status:**	
Living together	277 (69.6)
Separated	59 (14.8)
Single/Windowed/Divorced	40 (10.1)
Non response	22 (5.5)
**Father's Education**	
< Secondary School	67 (16.8)
≥ Secondary School	331 (83.2)
**Mother's Education**	
< Secondary School	82 (20.6)
≥ Secondary School	316 (79.4)
**Father occupation:**	
Unemployed	24 (6.0)
Artisan/Trader	165 (41.5)
Professionals	108 (27.1)
Others/Don't Know	101 (25.4)
**Mother's Occupation:**	
Unemployed	23 (5.8)
Artisan/Trader	253 (63.6)
Professional	64 (16.1)
Don't Know	38 (9.5)
Others	20 (5.0)

Table 2 summarizes the types and characteristics of sexual abuse among study participants. Of the 398 adolescents interviewed, 362 (91.0%) responded either yes or no to the question, “Any unwanted sexual experience? The remaining 36 (9.0%) declined to respond to the question. Ninety three (25.7%) of the 362 participants reported a history of sexual abuse. This consists of 17 (24.3%) of 70 males and 76 (26.0%) of 292 females. In majority of the abused respondents the abuse happened only once (63; 67.8%), and at age 9–12 years (42; 45.2%). There was penetrative sexual abuse in 30 (32.2%) of the abused children and forced sexual abuse (rape) occurred in 43 (46.2%) of the sexually abused respondents. The perpetrators were mainly boyfriends (29; 31.2%), neighbours (15; 16.1%), relatives (7; 7.5%), and strangers (7;7.5%). Other perpetrators included class teachers, school mates and domestic helps. The major types of sexual abuse experienced by the respondents included kissing (64.5%), touching of the child's private parts (62.4%), being made to watch pornographic materials (55.9%), and sexual intercourse (32.2%).

**Table 2:** History and characteristics of child sexual abuse

Variable	Frequency (%)
**Any Unwanted Sexual Experience (N=362):**	
Yes	93 (25.7)
No	269 (74.3)
**Age at First Sexual Abuse (N=93)**	
Can't remember	20 (21.5)
5–8 years	15 (16.1)
9–12 years	42 (45.2)
≥ 13 years	16 (17.2)
**Perpetrator of sexual Abuse:**	
Parent/Other Relative	7 (7.5)
Neighbor	15 (16.1)
Domestic help/Schoolmate	16 (17.2)
Boyfriends	29 (31.2)
Teacher	3 (3.3)
Stranger	7 (7.5)
Others	6 (6.5)
Non response	10 (10.7)
**Times Sexually Abused (N=93):**	
Once	63 (67.8)
Several times	30 (32.2)
**Place of Sexual Abuse (N=93):**	
Home	23 (24.7)
At school	22 (23.7)
Neighbor house	22 (23.7)
Other places	26 (27.9)
**Type of sexual abuse:**	
Kissing	60 (64.5)
Touching private parts	58 (62.4)
Show me their private parts	48 (51.6)
Showing pornographic magazine/films	52 (55.9)
Took pictures of me naked	7 (7.5)
Sexual intercourse:	30(32.2)
*(Vaginal)*	*23 (24.7)*
*(Anal)*	*7 (7.5)*
Made me have sex with another person	3 (3.2)

Table 3 shows the pattern of sexual abuse disclosure among the respondents. Among the 93 adolescents who reported sexual abuse, only 32 (34.4%) had disclosed the abuse. The disclosure was mostly to parents (68.8%) and occurred days to months after the incident (24; 75.0%). Majority of those who disclosed (43.8%) were taken to the hospital after disclosure and 9.4% were not believed. The main reasons for non-disclosure were feelings of shame (40.4%), belief that nothing would come from disclosing the incident (31.6%), fear of the abuser (10.5%) and not being believed (17.5%). Among the 32 respondents that disclosed, 43.8% were taken to hospital, nothing was done in 31.2% and the remaining were either not believed (9.4%) or scolded (6.2%).

**Table 3:** Disclosure of sexual abuse among the respondents

Variable	Frequency (%)
**Any Disclosure of Incident**	
Yes	32(34.4)
No	57( 61.5)
Non-response	4(4.3)
**Disclosed to**	
Father/ mother	22(68.8)
Sibling	8(25.0)
Teacher	2(6.2)
**Time of Disclosure**	
Immediately	8(25.0)
Days later	21(65.6)
Months later	3(9.4)
**Reason for Non-Disclosure**	
I was ashamed	23(40.4)
They would not believe	10(17.5)
I was afraid of the user	6(10.5)
Nothing would come of my telling	18(31.6)
**What Happened after Disclosure**	
Nothing	10(31.2)
You were not believed	3(9.4)
You were scolded	2(6.2)
You were taken to the hospital	14(43.8)
Others	3(9.4)

## Discussion

The present community based cross-sectional study set out to ascertain the burden of child sexual abuse among adolescents in Mushin, a densely populated, middle to low income community in Lagos State, in SouthWestern Nigeria. It specifically focused on adolescents because they are often neglected and do not receive attention even when abused in our setting. Highlighting their sexual abuse challenges hopefully will bring focus and attention to it.

Three hundred and ninety eight in-school and out-of-school adolescents aged 10 years to 19 years participated in the study. The mean age of participants was 15.6 ± 2.0 years showing that more of the participants were adolescents in their mid-teens. Majority of the participants were single and in the senior secondary school classes. This is expected as the senior secondary school system in Nigeria is attended by middle and late adolescents who comprised most of our study population. Also age at marriage in Southern Nigeria is generally about 5 years higher than in the Northern part of the country^20^ so it is not surprising that majority of the study participants were single.

The major reason for stopping school for the 63 participants who were not in school was financial constraints. This reflects the finding by Odeyemi and colleagues^13^ in a similar population in 2009 and is probably because the study was carried out in a medium to low socio-economic community. However it shows that not much has changed in terms of educational challenges of adolescents in the study setting and highlights the need to make education accessible and affordable for the less privileged in the society. In Lagos state, Nigeria, where the study was conducted, there are no school fees in public schools, however the other wrap-around fees/cost that will enable a child attend school like cost of school uniform, transportation and textbooks are still unaffordable by significant percentage of children from poor homes. It is not surprising that children drop out of school despite the “free public education”.

Most of the participants (89.4%) lived with parents or guardians. This is in consonance with findings from previous studies^13^ in similar settings and is in keeping with the fact that adolescence is still a period of dependence on parents or guardians to provide for their wellbeing. More participants (57.3%) lived in one- or two-room accommodation with shared conveniences, also reflecting the socio-economic setting of the study. Most of the parents were traders or artisans and were married and living together, as also found by Odeyemi *et al*^13^. This may reflect the fact that divorce is not yet very prevalent in our society. The study revealed a child sexual abuse prevalence of 25.7% among respondents. This agrees with several studies from both within Nigeria and outside the country which have shown varying prevalence of 3% to 38%.^3^–^13^. This shows that child sexual abuse is highly prevalent in our society and should be of public health concern. The prevalence from this study is however lower than the 69.9% reported among female hawkers in SouthEastern Nigeria by Ikechebelu *et al* in 2009. This discrepancy with their study is probably explained by the fact that our population is made up of a mixed group of both in-school and out-of-school adolescents who may not be as vulnerable to abuse as street hawkers.^14^ The high rate of sexual abuse among adolescent in this study and other studies in Nigeria highlight the poor state of implementation of existing laws and statue on sexual abuse in the country. Although a number of laws that addressed sexual abuse of minors or sexual offenses exist in different forms, the challenge is with the implementation. In addition, culturally parents are unwilling to prosecute offenders as they see it as exposing their abused child to the public. This in our society has implication for the victim's future marriage prospects. Government, child right advocates and practitioners need to educate parents on the danger of not reporting child sexual abuse as it may be an indirect signal for perpetrators to continue abuse of children. Prosecution and punishing offenders of sexual abuse has the potential to deter other would be offenders.^11^,^15^ To address the deficiencies of the previous laws and statues, Lagos state, Nigeria parliament in 2015 passed into law, the sexual Offences Bill 2015 (www.drcc.ie/2015/09/press-release-the-criminal-law.) . This bill stipulates life imprisonment for persons convicted of sexual violation of children aged 18 years and below. It is hoped that penalty for the offense of sexual violation will be deterrent to those who plan to abuse children. The law, good as it, has a challenge of implementation as in previous laws. However the bill did one important thing, it raised the awareness in child sexual abuse in our society. It is hoped that parents will seize the opportunity not only to report cases but take their abused wards to hospital for psychological and post exposure prophylactic therapy.

Penetrative abuse occurred in 7.5% of respondents and this is similar to the 5% found by Lalor^9^ in a review of child sexual abuse literature from across the African continent. The age of initiation of sexual abuse was between 9 years and 12 years in a higher proportion of the cases. This agrees with the landmark study by Finkelhor on child sexual abuse^5^ which found the age of initiation of abuse to be between 8 years and 12 years of age. This is probably explained by the fact that secondary sexual characteristics begin to emerge during this age group, bringing the children to the unwholesome attention of potential abusers, while they still remain very vulnerable to abuse as a result of their level of physical and intellectual development. Also the adolescents at this stage feel they are adult and want to be independent, without knowing that they are putting themselves in harm's way. The government, parents and practitioners have important roles to play during this transition by providing the right education, counselling services, social support and safe environment.

Majority (67.8%) of the sexual abuse happened only once. The reason for this is not very clear. However it may be because in our society morality still has a place and the sexual abuse of children is perceived with disgust. The abuser may have yielded to a flitting impulse (which the perpetrator perceived as wrong) when an opportunity presented itself, but thereafter decided not to repeat the act, It may also be because the perpetrators look for easy targets and move on to other vulnerable victims to minimize the chances of exposure. In addition, the adolescent victim may have learnt one or two lessons in not presenting him/herself in harm's way.

Place of sexual abuse was evenly spread among home, school, neighbours's house and others. This agrees with several authors who have found that child sexual abuse can take place in a variety of settings.^9^ The above findings are not unexpected as these are places where adolescents spend most of their time. It also highlights the need to make home and school safe for adolescent. Parents should not assume that may sending a child to school or leaving the child at home protects the child against sexual abuse. Government should mount public health education and enlightenment on the challenge of child sexual abuse, factors associated with child sexual abuse and the strategies to prevent it. Also child abuse and its prevention education should be introduced in school and colleges as a long term strategy to reduce the burden of child sexual abuse.

The types of abuse suffered varied from non-contact abuse including being made to watch pornographic movies, to contact sexual abuse including vaginal and anal sexual intercourse. This agrees with various studies showing a variety of types of child sexual abuse.^1^,^3^,^12^ The concurrence in this studies are not surprising as abuse usually starts first with non-contact sexual abuse and if not addressed on time eventually progresses to contact sexual abuse. It further shows that neither environment nor setting affects the pattern and nature of abuse.

This study showed the main perpetrators of sexual abuse to be boyfriends (31.2%) and neighbours (16.1%). Intra-familial abuse occurred in 7.5% of cases with incest making up 2.1% of cases. On the other hand sexual abuse by strangers occurred in 7.5% of cases. This buttresses the observation by many authors that child sexual abuse is usually perpetrated by someone known to, and trusted by the child.^1^,^9^,^21^,^22^ This is in contrast to reports from some Asian cities, notably Bangladesh and Japan, that strangers make up the majority of child sexual abuse perpetrators.^23^ This discrepancy may mean that environment or culture or both may influence perpetration of child sexual abuse. However, in our environment, to prevent child sexual abuse, government and all stakeholders in child welfare should bring to fore the contribution of persons known to the child to his/her sexual abuse. With this information parents and guardian will be cautious on who they leave their child with in the ward.

Forced sexual abuse i.e. rape, occurred in 46.2% of the cases of child sexual abuse in this study. This is contrary to findings by most authors that force or violence is rarely used in cases of child sexual abuse.^1^ It is also higher than the 13.1% found by Odeyemi and colleagues^13^ in a similar population some years ago. Their relatively low prevalence may be because they were looking at sexual experiences of adolescents in general, including consensual sexual intercourse and not just sexual abuse. However our prevalence is closer to the finding of 28.1% among female street hawkers in Eastern Nigeria.^14^ The high prevalence of rape in this study may be accounted for by the fact that participants were older adolescents who were unlikely to be deceived into quietly acquiescing to sexual abuse, and so were more likely to resist thereby necessitating the use of force. It may also be because those who had been forcefully abused were more likely to admit to the abuse than those who acquiesced meekly to the abuse. This later group may feel that they collaborated in their own defilement and so may not readily own up to the abuse.

This study revealed that among the 93 participants that were victims of child sexual abuse, only 32 (34.4%) had disclosed the abuse, most often to a parent (68.8%) or to a sibling (25.0%). This rate of disclosure is higher than the less than 25% generally reported,^3^ but is lower than the 69%–81% reported by Priebe and Svedin.^24^ They also report that disclosure was less likely in cases of contact sexual abuse or if the abuse occurred only once. The high rate of contact (at least 64.5%) and single-incident CSA in our study (67.8%) may explain why our disclosure rate is lower than theirs. However in agreement with other studies, immediate disclosure (in 8.6% of cases) was also low in this study.^3^ The low sexual abuse disclosure in this study may be due to the abused feeling that reporting the case will not yield any fruit rather a negative publicity. This may reflect the current situation in the country where sexual abuse legislation is not strictly enforced. Parents and guardian are also not willing to report because of the stigma and discrimination associated with sexual abuse. The public should be educated on the dangers of sexual abuse and not reporting the sexual abuse. In addition the identity of the abused child should be protected. The Sexual Offences Bill 2015 which has been passed into law in Lagos State stipulates life imprisonment for persons convicted of sexual violation (defilement) of children aged 18 years and below. This will hopefully not only have a positive impact on disclosure of abuse but also serve as a deterrent to potential offender.

A possible limitation of the study was possible biased answers from the respondents as rape and sexual abuse are stigmatizing sexual experiences. Also the fear of perpetrator especially if s/he is close may have influenced the response of the respondents. The refusal of 9.0% adolescent in the study to respond to the question, “Any unwanted sexual experience”, attests to the above. We tried to eliminate getting biased answers by only using trained interviewers who are conversant of how to elicit sensitive information.

The study has various strengths, one of which is that it was a community based one. This allowed for the estimation of the true population prevalence and pattern of child sexual abuse among adolescents. The community of the study is also unique as it can be described as representative of the almost the entire Nigeria geopolitics. Majority of the population resides in the rural part of the setting as is the case with the country. Also the diversity of ethnicity and socio-economic characterisitics is almost representative making generalization of the findings easier. The use of systematic random sampling and recruitment of respondents strengthens the findings further.

## Conclusion

The prevalence of adolescent sexual abuse in the study community is 27.5%, with majority of perpetrators being persons known to the abused child and only 34.4% disclosure rate. This highlights the burden, challenge of sexual abuse among adolescent in our setting and the non-implementation of existing laws in the country. Public health education, advocacy and an introduction of sexual abuse prevention education in schools and colleges is recommended to reduce the burden and ill health associated with child sexual abuse. Parents and wards should be educated on the dangers of child sexual and abuse and encouraged to report cases not only to serve as deterrent to abusers but to available the victim of opportunity to receive therapy.
